# Examining How Internet Users Trust and Access Electronic Health Record Patient Portals: Survey Study

**DOI:** 10.2196/28501

**Published:** 2021-09-21

**Authors:** Rong Yin, Katherine Law, David Neyens

**Affiliations:** 1 Department of Industrial Engineering Clemson University Clemson, SC United States; 2 Human Factors and User Experience Medtronic Mounds View, MN United States; 3 Department of Bioengineering Clemson University Clemson, SC United States

**Keywords:** internet, consumer health informatics, patient portal, participatory medicine, electronic health records, logistic model, surveys, questionnaires

## Abstract

**Background:**

Electronic health record (EHR) patient portals are designed to provide medical health records to patients. Using an EHR portal is expected to contribute to positive health outcomes and facilitate patient-provider communication.

**Objective:**

Our objective was to examine how portal users report using their portals and the factors associated with obtaining health information from the internet. We also examined the desired portal features, factors impacting users’ trust in portals, and barriers to using portals.

**Methods:**

An internet-based survey study was conducted using Amazon Mechanical Turk. All the participants were adults in the United States who used patient portals. The survey included questions about how the participants used their portals, what factors acted as barriers to using their portals, and how they used and how much they trusted other web-based health information sources as well as their portals. A logistic regression model was used to examine the factors influencing the participants’ trust in their portals. Additionally, the desired features and design characteristics were identified to support the design of future portals.

**Results:**

A total of 394 participants completed the survey. Most of the participants were less than 35 years old (212/394, 53.8%), with 36.3% (143/394) aged between 35 and 55 years, and 9.9% (39/394) aged above 55 years. Women accounted for 48.5% (191/394) of the survey participants. More than 78% (307/394) of the participants reported using portals at least monthly. The most common portal features used were viewing lab results, making appointments, and paying bills. Participants reported some barriers to portal use including data security and limited access to the internet. The results of a logistic regression model used to predict the trust in their portals suggest that those comfortable using their portals (odds ratio [OR] 7.97, 95% CI 1.11-57.32) thought that their portals were easy to use (OR 7.4, 95% CI 1.12-48.84), and frequent internet users (OR 43.72, 95% CI 1.83-1046.43) were more likely to trust their portals. Participants reporting that the portals were important in managing their health (OR 28.13, 95% CI 5.31-148.85) and that their portals were a valuable part of their health care (OR 6.75, 95% CI 1.51-30.11) were also more likely to trust their portals.

**Conclusions:**

There are several factors that impact the trust of EHR patient portal users in their portals. Designing easily usable portals and considering these factors may be the most effective approach to improving trust in patient portals. The desired features and usability of portals are critical factors that contribute to users’ trust in EHR portals.

## Introduction

Patient portals are websites or mobile apps that are designed to help patients access their electronic health records (EHRs), health summaries, pay bills, schedule appointments, and, in some cases, interact with care providers [1]. The use of patient portals has been associated with generating positive health care outcomes in recent studies [2,3]. For example, individuals and families have been shown to be more actively engaged in their health management [4] with better information communication [5]. Using EHR portals may also contribute to increasing the efficiency and effectiveness of health care providers [2].

The US government has been promoting the use of patient portals through federal laws such as the Health Information Technology for Economic and Clinical Health Act of the American Reinvestment and Recovery Act [6], which requires that health providers prove the usefulness of EHRs (defined as using EHRs in a meaningful manner) through a three-stage process [7]. The “meaningful use” of EHR portals is believed to have a positive impact on improving the quality of health care [8]. Government promotion was suggested as one of the major reasons for providers to encourage their patients to register on EHR portals despite the positive benefits of EHR portals [5]. Owing to these requirements, the adoption of EHRs in hospitals increased from 9% in 2008 to 80.5% in 2015 [9].

Although some research has shown a potential correlation between low health literacy and a lower likelihood of using patient portals, the results are inconsistent across studies [10-12]. Meanwhile, vulnerable patients may also require that portals have higher usability (eg, portals that are easy to use) and intensive training may be necessary in such cases [13]. Demographic characteristics such as gender, education, and income have been shown to impact the EHR usage rates [14]. Additionally, other barriers such as the digital divide, and concerns related to privacy and data security have also been shown to impact EHR usage rates [15]. A recent study suggested that the use of EHR portals is still low, although it has been increasing (from 25.6% to 31.4% between 2014 and 2018 according to the data of the Health Information National Trends Survey [14]). Addressing the barriers associated with using portals may not only increase the usage rate of patient portals but may also contribute to improving patients’ trust in their providers, thus encouraging patient-provider communication [16] and potentially improving patient health outcomes. Younger adults and individuals who trust the internet more could have an easier time using patient portals [17]. Additionally, patients who highly trust their health care providers are more likely to use their portals [16]. Generally, most of the trust-related studies about EHR portals focus on the patient-provider relationship [4,18,19]. Few studies have analyzed the patients’ trust in the EHR portals themselves. Studies in similar domains (such as trust in health information websites) have shown the importance of trust in determining which websites to use and how to best use their content [20]. Trust in health websites, such as EHR portals, is an important factor to examine as the internet is not considered a fully reliable source of health information [20,21].

Few studies have focused on the factors that impact patients’ trust in EHR portals. A scoping review [22] of multiple studies recommended that specifying the features of EHR portals for certain primary care patient groups was necessary. Thus, the purpose of our study was to conduct an internet-based survey to examine how current portal users report using their patient portals and the factors impacting their trust in their portals. To better examine how current portal users use and trust their EHR portals, we also examined how they access health information and trust those information sources, and what design features of these patient portals are preferable for continued use.

## Methods

### Survey Design

Our internet-based survey was designed using Qualtrics and distributed using Amazon Mechanical Turk. Amazon Mechanical Turk is a widely used [23] internet-based tool to recruit people to perform virtual tasks such as survey participation and content moderation. Many participants can be recruited efficiently using Amazon Mechanical Turk [24]. The data obtained using Amazon Mechanical Turk have been considered reliable [25-27] and more representative of the general population [28] than the data obtained from convenience samples (eg, college students) and generally represent diverse backgrounds [29].

The survey (see Multimedia Appendix 1) was designed with specific questions for patient portal users, and a slightly different version of the survey was used if participants reported that they were not current portal users. We included a wide variety of questions in the survey to assess the perceptions of participants on patient portals, how they are accessed and used, difficulties in using patient portals (eg, data safety and security and difficulty in understanding information presented in the portal), and what features are desired in these portals. The survey also contained questions about seeking, accessing, and trusting health information from other sources. We included specific questions to evaluate how much portal users trust their current portals. Participants were asked to respond to a 5-point Likert scale (ranging from “strongly agree” to “strongly disagree”) to the following statement: “I trust the current EHR portals that I am using.”

None of the questions required mandatory responses and we also included options such as “Do not know” or “Prefer not to answer” for some questions, as appropriate. We also included free response options for some questions. Two quality check questions were included in the survey to ensure that the participants were answering the questions carefully rather than randomly choosing an answer (eg, we asked the participants to choose “yes” for a subitem of a question and asked them to select “strong agree” for another question). We removed the responses of the participants who did not answer the quality check questions correctly.

### Participants

The participants were required to be residents living in the United States aged over 18 years. We recruited 500 participants to participate in the survey. After removing the participants (46 participants) who failed to answer our quality check question in the survey and those who were not EHR portal users (60 participants), we included 394 participants in this analysis, who were current portal users. This study was identified as a research activity involving human subjects that met exemption criteria under the Code of Federal Regulations (CFR), namely 45 CFR 46 and 21 CFR 56 by the Clemson University Institutional Review Board, as the survey was anonymous, and no identifiable data were collected. The data were collected in January 2020. Each participant received US $1 as compensation for completing the survey using Amazon Mechanical Turk.

### Data Analysis

Simple statistics were used to describe the survey population along with several different parameters. In our data analysis, some of the subjective rating questions that used 5-point Likert scale options were converted to binary answers. For example, the Likert scale options of “agree” and “strongly agree” were combined into a single category that was compared to all other Likert scale responses. Logistic regression was used to explore what factors impacted portal users’ trust in their portals. We used the stepwise Akaike Information Criterion (AIC) selection method [30] to identify the best fit model. We performed an automated AIC forward stepwise selection procedure using the StepAIC function in the Modern Applied Statistics with S package in R (version 4.0.2). This function automatically adds variables into a model such that the AIC is lower with the additional variable than without it. This function identifies the variable set that produces a model with the lowest AIC value among all the possible variables. We included 13 explanatory variables in the final model after applying this AIC selection method. We set α=.05 as the level of statistical significance. The data analysis was conducted using R (R Foundation for Statistical Computing).

## Results

### Descriptive Statistics

#### Demographics

Approximately half of the participants (212/394, 53.8%) were younger (less than 35 years old), followed by 36.3% (143/394) that were middle-aged (35-55 years old), and 9.9% (39/394) that were older (over 55 years old), as observed in Table 1. Female portal users accounted for 48.5% (191/394) of our participants. Almost all the participants (372/394, 94.4%) reported being employed and most (372/394, 94.4%) of the participants reported being covered by a health insurance plan. Additionally, 72.6% (286/394) of our participants had their most recent health care appointment within the last 6 months.

Overall, 23.9% (94/394) of the participants reported using EHR portals weekly or more frequently, whereas 46.7% (184/394) reported having used their portals monthly and 29.4% (116/394) of the participants reported using their portals only yearly or less often. Furthermore, 48.7% (192/394) of the participants reported sending messages through the EHR portals to their care providers annually or more frequently. Meanwhile, 54.3% (214/394) of the participants reported receiving messages through the EHR portals from their care providers at least annually.

**Table 1:** Characteristics of participants who are current portal users (N=394).

Characteristic		Participants, n (%)
**Age**		
	Younger adults (<35 years)	212 (53.8)
	Middle-aged adults (from 35 to 55 years)	143 (36.3)
	Older adults (>55 years)	39 (9.9)
**Gender**		
	Male	203 (51.5)
	Female	191 (48.5)
**Education**		
	Educated to high-school level or lower	38 (9.6)
	Some college or graduate education	356 (90.4)
**Income**		
	Less than US $52,000	222 (56.3)
	More than US $52,000	172 (43.7)
**Marital status**		
	Married	251 (63.7)
	Not married	143 (36.3)
**Employment status**		
	Employed	372 (94.4)
	Unemployed	12 (3)
	Retired	10 (2.5)
**Internet use frequency**		
	At least daily	368 (93.4)
	Less than daily	26 (6.6)
**Insurance status**		
	Insured	372 (94.4)
	Uninsured	22 (5.6)
**Last health care appointment**		
	Less than 6 months	286 (72.6)
	More than 6 months	108 (27.4)
**Portal use frequency**		
	Weekly or more frequently	94 (23.9)
	Monthly	184 (46.7)
	Yearly or less	116 (29.4)
**Message exchange**		
	Send messages to providers annually or more frequently	192 (48.7)
	Received messages from providers annually or more frequently	214 (54.3)

#### Participants’ Views of Their Portals

Most of the participants (300/394, 76.1%) consider their portals as a valuable part of their health care, with 93.4% (368/394) of the participants believing that their portals were easy to use. Overall, 76.6% (302/394) of the participants reported that they believed using portals had become habitual in managing their health. Additionally, most of the participants (366/394, 92.9%) reported trusting their portals, and 90.4% (356/394) of the participants reported believing that their portals were important in managing their health. Furthermore, 93.4% (368/394) of the participants thought that it was important to have a record of past health information (eg, visit history, lab results, and appointments) on their EHR portals. A total of 92.4% (364/394) of the participants reported that they were comfortable with their portals.

#### Portal Features Used by Participants

The participants could choose multiple answers that fit their conditions. There were primarily 10 features that were used by portal users, as shown in Table 2. The most frequently used features of portals were “view lab results” (229/394, 58.1%), “make/check appointments” (215/394, 54.6%), and “view/pay bills” (201/394, 51%). Approximately half of the participants (195/394, 49.5%) reported using portals to check their visit history. Meanwhile, 33.3% (131/394) of the participants reported using their portals to contact their health providers, and 27.4% (108/394) of the participants reported having requested prescription refills through portals. Only a few participants had used other features including educational materials (54/394, 13.7%), immunization reports (41/394, 10.4%), and review allergies and alerts (33/394, 8.4%).

**Table 2:** Portal features used by participants (N=394).

Portal feature	Participants, n (%)
View lab results	229 (58.1)
Make and check appointments	215 (54.6)
View and pay bills	201 (51)
Check my visit history	195 (49.5)
Contact my health providers	131 (33.3)
Prescription refill request	108 (27.4)
Medications	83 (21.1)
Educational materials	54 (13.7)
Immunizations	41 (10.4)
Document and review allergies and alerts	33 (8.4)

#### Factors Leading to Difficulty in Using Portals

The survey included questions about what design features or factors led to difficulty in using patient portals. The most frequently reported factors that made portals difficult to use were concerns about data safety and security (136/394, 34.5%), as indicated in Table 3. Some (111/394, 28.2%) participants reported limited access to the internet as a factor that led to difficulty in using portals. Irrelevant messages (88/394, 22.3%) and being unable to view enough patient information (81/394, 20.6%) were the other two leading factors that made portals difficult to use. As common issues with most web-based products, spam and too many messages (55/394, 14%) and lost passwords (51/394, 12.9%) were also noted to result in difficulties. Difficulty in understanding the health information on their patient portals was reported by 11.7% (46/394) of the participants, whereas only 3.3% (13/394) of the participants reported that they did not trust the information displayed on the patient portals. Additionally, 7.6% (30/394) of the participants reported preferring to use other websites (eg, WebMD, Wikipedia, and Google) rather than their portals.

**Table 3:** Factors causing difficulty in using portals as reported by participants (N=394).

Factor	Participants, n (%)
Concerns about my data safety and security	136 (34.5)
Limited access to the internet	111 (28.2)
Messages that are not relevant to me	88 (22.3)
Unable to view enough patient information	81 (20.6)
Spam and too many messages	55 (14)
Lost password	51 (12.9)
Difficult to understand the information in portals	46 (11.7)
Preference for other websites instead (eg, WebMD, Wikipedia, and Google)	30 (7.6)
Not trusting the information displayed	13 (3.3)

#### Sources of Health Information

The participants were asked whether they had ever used other online information sources to obtain health information, and they could choose multiple answers. As seen in Table 4, most participants (331/394, 84%) reported having used WebMD for health information. Internet-based medical articles were used by 76.4% (301/394) of the participants and Wikipedia was used by 68% (268/394). More than half of the participants (221/394, 56.1%) reported having used health blogs to obtain health information. Approximately half of the participants reported using government and hospital websites to obtain health information. Meanwhile, some of the participants also reported using social media platforms such as Facebook (128/394, 32.5%), Twitter (106/394, 26.9%), and Instagram (98/394, 24.9%) to access health information.

**Table 4:** Online information sources that participants used to obtain health information (N=394).

Source	Participants, n (%)
WebMD	331 (84)
Internet-based medical articles	301 (76.4)
Wikipedia	268 (68)
Health blogs	221 (56.1)
Government websites	200 (50.8)
Hospital websites	200 (50.8)
Facebook	128 (32.5)
Twitter	106 (26.9)
Instagram	98 (24.9)

Across several internet-based sources of health information, WebMD and medical articles were reported as the most frequently trusted health information sources, with 79.2% (312/394) and 77.9% (307/394) of our respondents reported trusting WebMD and internet-based medical articles, respectively, as observed in Table 5. Hospital system websites and government websites were also highly trusted, with 75.6% (298/394) and 68.3% (269/394) of the participants trusting the sources, respectively. Although 68.3% of the participants used Wikipedia for health information, only 59.1% (233/394) trusted it. Health blogs were also trusted by more than half of the participants (215/394, 54.6%). Other social media platforms such as Facebook (108/394, 27.4%), Twitter (99/394, 25.1%), and Instagram (99/394, 25.1%) were trusted by fewer participants than the other information sources.

**Table 5:** Internet-based sources of health information sources that participants reported trusting (N=394).

Source	Participants, n (%)
WebMD	312 (79.2)
Medical articles	307 (77.9)
Hospital websites	298 (75.6)
Government websites	269 (68.3)
Wikipedia	233 (59.1)
Health blogs	215 (54.6)
Facebook	108 (27.4)
Twitter	99 (25.1)
Instagram	99 (25.1)

#### Information Presentation Method

The participants were asked to identify their preferences for the presentation of health educational materials and could choose multiple answers. Most of the participants (250/394, 63.5%) believed that videos were the most effective way to present health educational materials, followed by texts (196/394, 49.8%), photographs (126/394, 32%), and diagrams or charts (105/394, 26.7%).

#### Accessing EHR Patient Portals

Approximately half of the participants (184/394, 46.7%) reported using their EHR portals monthly, 23.1% (91/394) reported using EHR portals on a yearly basis, and 19% (75/394) used their portals weekly. Meanwhile, daily portal usage was reported by 4.8% (19/394) of the participants. Only 6.4% (25/394) of the participants reported using their patient portals only once.

In terms of how the participants accessed their portal, most participants (305/394, 77.4%) used their portals through home computers (the participants could select more than one option). The other two common EHR portal access approaches were mobile devices (118/394, 28.9%) and work computers (95/394, 24.1%). Very few participants reported using EHR portals through public computers such as library computers (14/394, 3.6%) and school computers (4/394, 1%).

#### Contacting Health Providers With Questions

We assessed how many participants used secure messaging through their portals to contact their health care providers. The participants reported that “messages through portals” constituted the most (156/394, 39.6%) used method to contact their health care providers. Another widely reported method for contacting their health care providers was through telephone (146/394, 37.1%). Meanwhile, only 12.9% (51/394) and 9.9% (39/394) of our participants, respectively, reported using email or scheduling an in-person visit when they had health-related questions for their health providers.

### Predicting Users’ Trust in the EHR Patient Portal

We built a logistic regression model to predict the EHR portal users’ trust in their patient portals, as shown in Table 6. Compared to others, participants who were frequent internet users (ie, used the internet at least daily) were significantly more likely to trust their portals (odds ratio [OR] 43.72, 95% CI 1.83-1046.43). Participants who were comfortable using their EHR portals were more likely to trust the portals that they were currently using (OR 7.97, 95% CI 1.11-57.32). Participants who believed their portal was important in terms of managing their health (OR 28.13, 95% CI 5.31-148.85) or who believed that their EHR portal was a valuable part of their health care (OR 6.75, 95% CI 1.51-30.11) were more likely to trust their portals. Participants who used Wikipedia (OR 12.87, 95% CI 2.23-74.26) or social media platforms (such as Facebook, Twitter, and Instagram; OR 4.44, 95% CI 1.14-17.24) for obtaining health information were also more likely to trust their EHR portals. Meanwhile, the participants’ trust in some web-based health information sources was positively related to the trust in their portals. Participants who trusted WebMD (OR 3.98, 95% CI 1.11-14.32) or government websites (OR 7.73, 95% CI 1.92-31.19) to obtain health information were also more likely to trust their EHR portals. Some factors that led users to believe that their portals were difficult to use were negatively associated with the participants’ trust in their portals. Participants who believed that they received irrelevant messages (spam or too many messages) through their portals were less likely to trust their portals (OR 0.05, 95% CI 0.005-0.61). In contrast, participants who found their portals easy to use were more likely to trust their portals (OR 7.4, 95% CI 1.12-48.84).

**Table 6:** Logistic regression model to predict users’ trust in electronic health record portals.

Factor			Estimate		SE		Z value		*P* value		Odds ratio (95% CI)
Intercept			–12.21		2.54		–4.81		<.001		—^a^
Comfortable in using my EHR^b^ portal			2.08		1.01		2.06		.04		7.97 (1.11-57.32)
EHR portal is important in managing my health			3.34		0.85		3.92		<.001		28.13 (5.31-148.95)
Used Wikipedia for health information			2.56		0.89		2.86		.004		12.87 (2.23-74.26)
Trust WebMD to get health information			1.38		0.65		2.12		.03		3.98 (1.11-14.32)
Spam made my portal hard to use			–2.94		1.25		–2.36		.02		0.05 (0.005-0.61)
Trust government websites			2.05		0.71		2.88		.004		7.73 (1.92-31.19)
EHR portal is a valuable part of my health care			1.91		0.76		2.50		.01		6.75 (1.51-30.11)
Hard to understand information in my portal			–2.03		1.10		–1.84		.07		NS^c^
Irrelevant message made my portal hard to use			–1.02		0.82		–1.24		.22		NS
Frequent internet users (daily use)			3.78		1.62		2.33		.02		43.72 (1.83-1046.43)
Used social media to get health information			1.49		0.69		2.16		.03		4.44 (1.14-17.24)
It is easy to use my EHR portal			2.00		0.96		2.08		.04		7.40 (1.12-48.84)
Older adults			–1.62		1.17		–1.39		.17		NS
**Model statistics parameters**											
	Likelihood ratio test result –2 log likelihood, *x*^2^ (*df*)	–84.64 (14)		N/A^d^		N/A		N/A		N/A	
	Model *P* value	<.001		N/A		N/A		N/A		N/A	
	χ^2^ (*df*)	117.396 (13)		N/A		N/A		N/A		N/A	
	AIC^e^	112.64		N/A		N/A		N/A		N/A	

^a^Not available.

^b^EHR: electronic health record.

^c^NS: no statistically significant differences found at α=.05.

^d^N/A: not applicable.

^e^AIC: Akaike Information Criterion.

#### Features That Would Encourage Future Portal Use

In addition to assessing the participants’ evaluation of their current patient portals, the participants were also asked about features (or potential features) that would encourage them to use their portals more. This question had 29 options that we provided based on the features identified in the literature or features that may potentially fit within an EHR patient portal (eg, mental health self-assessment). Participants were also able to include additional features that were not listed, and these might lead them to use their EHR portal more. The participants were able to select unlimited potential portal features that might encourage them to use the system more. Among all the features, more than one-third of the participants agreed that they would use their portals more if the portals included real-time chats with physicians, safe and secure messaging, and prevention and follow-up reminders, as observed in Table 7. Other features including real-time virtual appointments, lab results, and appointment requests were also important factors that might lead to increased portal use.

**Table 7:** Electronic health record patient portal features that participants reported wanting (N=394).

Factor	Participants, n (%)
Real-time chat with physicians	154 (39.1)
Safe and secure messaging	151 (38.3)
Reminders: preventive and follow-up	135 (34.3)
Real-time virtual appointment	126 (32)
Lab results	124 (31.5)
Appointment requests	121 (30.7)
Access materials (eg, lab reports, bills, or educational materials)	119 (30.2)
Prescription refill requests	119 (30.2)
Appointment reminders	103 (26.1)
Billing	99 (25.1)
Diagnostic test results	90 (22.8)
Insurance information	80 (20.3)
Patient-specific educational materials and web resources	77 (19.5)
Wellness and preventive care	74 (18.8)
Medications	66 (16.8)
Appointment log	65 (16.5)
Exercise information	65 (16.5)
Virtual therapy	64 (16.2)
Mental health resources and education	59 (15)
Mental health self-assessment	52 (13.2)
Immunizations	48 (12.2)
Problems lists	47 (11.9)
Calorie calculator and diet manager	44 (11.2)
Smart watch or Fitbit data entry	42 (10.7)
Public health information	40 (10.2)
Self-monitoring data entry	38 (9.6)
Allergies and alerts	32 (8.1)
Sexual health information	28 (7.1)

## Discussion

### Principal Findings

This study sought to investigate how individuals accessed health information and their EHR patient portals as well as identify barriers and facilitators for portal use. We conducted an internet-based survey that asked EHR portal users about their behaviors associated with portal usage, as well as their opinions about portal usage and about current and potential features of EHR portals. In general, most participants reported that their patient portals were valuable and that they trusted their portals.

Our results suggest that many factors contribute to users’ trust in EHR portals. The usage and trust associated with some other internet-based health information sources were also found significant in predicting the likelihood of patients trusting the portals. In contrast, spam, irrelevant messages, and difficult-to-understand information within the portals were identified as factors that could lead to a decrease in the likelihood of users trusting EHR portals. Thus, there are ways to design and manage future EHR systems that support patients to develop trust in their EHR portals. For example, when it is necessary to refer to a piece of educational health information (such as the definition, detection, and symptoms of hypertension) in EHR portals, referring to a trusted information source such as WebMD may potentially increase users’ trust in EHR portals. This is consistent with the research findings indicating that health care providers, the internet, and government health agencies are the three most trusted health information sources [31]. One study suggested that approximately one-third of the patients reported having difficulties in finding health information and concerns about the information quality [31]. Thus, providing necessary health information within EHRs has its potential value, and choosing a trusted health information source as a reference is vital in designing a trustworthy EHR. Ensuring that the EHR portals are easy to use and have easy-to-understand information may contribute to increased trust in these portals [32]. It is critical that users trust their EHR patient portals as well as the information and instructions contained in these portals; otherwise, the systems may not be valuable to the patients [33]. Moreover, patient trust in eHealth features including health websites is an important factor leading to crucial patient outcomes [34,35]. Identifying the factors and groups that have high trust and those who do not trust EHR patient portals can lead to better designed systems for users and increased trust in the EHR portals, which can eventually improve the use of EHR portals [32].

Generally, our sample of portal users included more younger and middle-aged adults, which is consistent with the population of EHR portal users in other survey studies [36,37]. We did not detect gender differences in the survey participants across our analyses. However, other studies have shown gender differences in terms of the access and use of EHR portals [37]. The use of EHR patient portals among more specific gender and age groups for specific diseases should be examined to reveal the specific user needs and characteristics, such as individuals having multiple chronic conditions who may need closer monitoring on their EHR portal [38]. Not everyone reported having access to fast and reliable internet connections, and there are populations of potential EHR portals users who were not represented in our survey sample. Thus, our survey participants reflect users with access to the internet and may not represent all the potential users of EHR portals.

Several studies have proposed improving self-health management through mobile health apps [39], and the integration of mobile apps with computer-based EHRs has been demonstrated [33,40]. Future studies should examine the factors related to internet characteristics in different locations (eg, home, public, or work) or on different platforms (eg, mobile, tablet, or computer). Designing EHR patient portals with effective displays for computers and mobiles may make the design of EHR portals more complex and introduce additional usability issues. Furthermore, our study suggests that most EHR users used their portals infrequently, such as monthly. Thus, the design of EHR portals needs to support easy learning and the ability to retain the knowledge about how to engage with the system.

Consistent with a previous study [41], data security concerns and limited internet access are the most frequent barriers that our participants reported as related to perceiving portals to be difficult to use, which was followed by irrelevant messages and being unable to view enough patient information. Future EHR portals designers should pay special attention to address security concerns, avoid irrelevant messages such as advertising messages, and provide comprehensive health information.

It has been shown that older adults have many potential barriers in using EHR portals such as limited health literacy, limited access to health technology, and preference for in-person communication [42,43]. Limited access to the internet and limited ability to use computer-based EHR technology were reported as some of the major barriers for elderly people to use EHR portals [41]. However, modern health technology features such as EHR portals may potentially provide significant benefits for specific groups of people with specific clinical needs. For example, there may be substantial benefits for the elderly, who may need to track their health records more frequently owing to multiple complex health conditions [43,44]. It is necessary for future research studies to specifically target groups of patient portal users (eg, older individuals and individuals with specific health conditions) and nonusers. A recent study suggests that some interventions (eg, an intervention that used one-on-one training on EHRs [45]) could improve EHR portal usage among vulnerable populations [46]. Future studies may examine EHR portal usage among different age groups with different internet accessibility levels, as well as interventions to promote the use of EHR portals.

Although secure messaging through EHR portals is believed to have a positive impact on patient-provider communication [47], the overall message communication between portal users and health providers was reported as infrequent in our study (less than half of the participants send messages through portals annually or more frequently, although slightly more participants received messages through portals). The communication through portals between patients and providers did not replace traditional communication approaches such as email, telephone, or text messaging. We could see that emerging methods like text messaging through EHR portals and traditional methods like telephone calls are commonly used when our participants had questions for their health care providers. Although health care providers believe that the use of EHR portals can positively impact information delivery and improve patient-provider communication according to a recent study [44], EHR portals are still not widely used for communication, and there are several opportunities to improve messaging features.

In general, there is no comprehensive understanding of how users feel about their patient portals and what factors are associated with their usage. Our study suggested that viewing lab results, checking appointments, and paying bills are the most commonly used portal features and the specifics of how these functions are designed and implemented is an important direction for future research. The features that are widely used and valued are the core features of patient portals. There are other features that participants want to use or those that would lead them to use their portals more often. For example, the ability to engage in real-time chats with care providers is an uncommon feature for most EHR portals, but our study demonstrated that is a highly valued and desired feature. This feature could help patients connect to their clinicians without always requiring an office visit, which would help reduce the burden on clinics while also providing individualized care. Additionally, reducing clinical visits when not necessary is critical during periods with highly infectious diseases (eg, COVID-19 or the annual flu season). Under the special situation of the COVID-19 pandemic, minimizing unnecessary in-person visits and conducting remote discussions are particularly valuable [48-50]. Based on the results of this study, these features may further encourage the use of EHR systems and help patients remain connected to their health care providers. Another web-based communication feature, namely safe and secure messaging, was also highly ranked by EHR portal users. In fact, among the top 10 desired features in our results, 4 were related to documentation (eg, lab results and billing), 3 to communication with health care providers, and 3 to appointments and scheduling such as appointment reminders and requests. Thus, there is value in continuing to develop tools for internet-based communication between EHR portal users and their care providers.

Our study was conducted within the United States, and thus the results are most relevant within the US health system. Although there are some features that are more universal and may apply to health systems across the world, some specific features related to billing are specific to the United States. Further, only 60 nonusers participated in the survey, and thus we did not include nonusers in the analysis. A separate study with a larger sample size of nonusers that examines the specific barriers for nonusers and their perspectives on EHR portals will contribute to the literature.

Our study also examined the methods that the survey participants reported preferring for the presentation of educational health information. Most of the participants preferred videos, which topped the other methods of information presentation. Written text (or using words) was ranked second and was viewed as a better way than photographs or diagrams and charts. Future studies should evaluate these preferences and determine how best to present information in multimodal strategies. Additionally, as videos were reported as the most preferred information presentation method, future research should examine what types of health information can be presented in the video format. Future research should also examine how video presentation impacts the comprehension of health information, considering how the design of video presentations may facilitate the information exchange process and improve communication efficiency. Videos have been shown to be effective for online education and do not require reading abilities and facilitate repeated viewing for comprehension; they may support different learning styles and lead to better learning outcomes [51,52].

Against the backdrop of the COVID-19 pandemic, the close monitoring of patients’ health conditions in a virtual or web-based modality is important for public health. For example, employers may require regular negative COVID-19 test results for in-person work, and thus, more people may be accessing and engaging with their EHR patient portal to access these test results. Therefore, frequent, safe, and easy access to their test results (eg, lab results section) is a critical design feature for the use of EHR patient portals. Special attention should be paid to design these features to satisfy the user needs and expectations; thus, future research should examine how to design and implement these types of features and specific features that are important for future portal users.

### Conclusion

This study examined the use of EHR portals by internet users. Our study provides insights into some desired features and factors that lead to users trusting their EHR patient portals. Additionally, we identified some of the frequently encountered barriers to using EHR patient portals. It should be noted that the survey was administered prior to the COVID-19 pandemic, and thus, it may not reflect current trends in the availability and use of internet-based health information and virtual health care appointments. In conclusion, designing effective and easily usable EHR portals may be the most effective approach to improving users’ trust in the portals. The features and interface design of EHR portals are critical factors that contribute to increasing users’ trust in EHR portals. Future work should evaluate how to most effectively design these features to extend the benefits of using EHR patient portals for monitoring health.

## Appendix

Multimedia Appendix 1 Survey on electronic health record patient portals created for and used in this study.

## Abbreviations

AIC: Akaike Information Criterion
CFR: Code of Federal Regulations
EHR: electronic health record
OR: odds ratio
