# Supplementary material for: Examining How Internet Users Trust and Access Electronic Health Record Patient Portals: Survey Study
Source: JMIR Hum Factors. 2021 Sep 21;8(3):e28501. doi: 10.2196/28501 (PMC8493465; doi:10.2196/28501)
Supplement: Multimedia Appendix 1 [file humanfactors_v8i3e28501_app1.docx]

A Survey about Patient Portals for Electronic Health Record

1. How often do you use your Electronic Health Record (EHR) portal?

1. Daily
2. Weekly
3. Monthly
4. Yearly
5. Only once
6. Never

2. Why do you use the EHR portals? (check all that apply)

1. Check my visit history
2. View/Pay bills
3. Make/check appointments
4. View lab results
5. Contact my health providers
6. Educational materials
7. Prescription refill request
8. Document/review allergies and alerts
9. Medications
10. Immunizations
11. Other, please specify:

3. How often have you sent a message through the EHR portals to your doctor or nurse?

1. Daily
2. Weekly
3. Monthly
4. Yearly
5. Never

4. How often have you received a message through the EHR portals from your doctor or nurse?

1. Daily
2. Weekly
3. Monthly
4. Yearly
5. Never

5. Generally, how do you access your EHR portal? (check all that apply)

1. Home computer
2. Work computer
3. Library computer
4. School computer
5. Mobile device/cell phone
6. Other, please specify:

6. Please respond with how much you agree or disagree with the following statements:

|  | Strongly agree | Somewhat agree | Neither agree nor disagree | Somewhat disagree | Strongly disagree |
| --- | --- | --- | --- | --- | --- |
| It is easy to use my EHR portal. |  |  |  |  |  |
| Using the EHR portal has become a habit for me now. |  |  |  |  |  |
| The EHR portal is useful in helping me to find the information I needed. |  |  |  |  |  |
| The EHR portal helps me make decisions about my healthcare needs. |  |  |  |  |  |
| Please choose “strongly disagree” for this statement. |  |  |  |  |  |
| I prefer that my doctor or nurse contact me via EHR portal rather than via phone call. |  |  |  |  |  |
| I prefer to receive messages from my doctor or nurse through my EHR portal rather than paper mails. |  |  |  |  |  |
| I prefer my doctor or nurse to send me messages through an EHR portal rather than emails. |  |  |  |  |  |
| Reading my doctor's or nurse's messages in my EHR portal helps me in understanding my health and medical conditions. |  |  |  |  |  |
| It is important to have a record of past health information (visit, lab results, appointments) on my EHR portal. |  |  |  |  |  |
| I trust the current EHR portals that I am using. |  |  |  |  |  |
| I am comfortable with my EHR portal. |  |  |  |  |  |
| The EHR portal is important in managing my health. |  |  |  |  |  |
| The EHR portal is a valuable part of my health care. |  |  |  |  |  |

7. How long did it take you to become familiar with your EHR portals?

1. The first time I used the EHR portal
2. Less than a day
3. A day to a week
4. A week to one month
5. More than a month
6. I am not familiar with EHR portals

8. How do you like to contact your doctor or nurse when you have questions?

1. Messages through portals
2. Email my health providers
3. Call my health providers
4. Schedule an in-person visit
5. Other, please specify:

9. I would use my EHR portal more if it had these features: (check all that apply)

1. Safe and secure messaging
2. Reminders: preventive and follow-up
3. Access or download materials (for example, lab reports, bills or educational materials)
4. Patient-specific educational materials and web resources
5. Prescription refill requests
6. Appointment requests
7. Appointment reminders
8. Allergies and alerts
9. Medications
10. Immunizations
11. Lab results
12. Diagnostic test results
13. Problems lists
14. Appointment log
15. Insurance information
16. Billing
17. Real time virtual appointment
18. Real time chat with physicians
19. Wellness/preventive care
20. Exercise information
21. Calorie calculator/diet manager
22. Self monitoring data entry
23. Smart watch or Fitbit data entry
24. Mental health resources and education
25. Mental health self assessment
26. Virtual therapy
27. Sexual health information
28. Public health information
29. Other, please specify:

10. What makes your EHR portals hard to use? – check all that apply:

1. Limited access to the internet
2. Unable to view enough patient information
3. Concerns about my data safety and security
4. Spam and too many messages
5. Messages that are not relevant to me
6. I prefer to use other websites instead (WebMD, Wikipedia, Google, etc.)
7. It is hard for me to understand the information in portals
8. I do not trust the information displayed
9. Lost password
10. Other, please specify:

11. What do you think is the most effective way to present health educational materials? (check all that apply)

1. Videos
2. Words
3. Photos
4. Drawings or charts

12. Do you think that a user guide or user manual would help you when using portals?

1. Yes
2. No
3. Don't know

13. Have you ever used any of these online information sources to get health information?

|  | Yes | No | Don't know |
| --- | --- | --- | --- |
| WebMD |  |  |  |
| Wikipedia |  |  |  |
| Online medical articles |  |  |  |
| Your EHR Portal |  |  |  |
| Choose 'Yes' for this one |  |  |  |
| Government websites |  |  |  |
| Hospital website |  |  |  |
| Health blogs |  |  |  |
| Facebook |  |  |  |
| Twitter |  |  |  |
| Instagram |  |  |  |

14. How much do you agree with the following statements: I trust the following online information resources for health information:

|  | Strongly agree | Somewhat agree | Neither agree nor disagree | Somewhat disagree | Strongly disagree |
| --- | --- | --- | --- | --- | --- |
| WebMD |  |  |  |  |  |
| Wikipedia |  |  |  |  |  |
| Online medical articles |  |  |  |  |  |
| Your EHR Portal |  |  |  |  |  |
| Government websites |  |  |  |  |  |
| Hospital website |  |  |  |  |  |
| Health blogs |  |  |  |  |  |
| Facebook |  |  |  |  |  |
| Twitter |  |  |  |  |  |
| Instagram |  |  |  |  |  |

15. If your doctor or nurse provides alternatives to meeting in person, would you be willing to have a:

|  | Yes | No | Don't know |
| --- | --- | --- | --- |
| Telephone appointment |  |  |  |
| Webcam video chat |  |  |  |
| Text chat |  |  |  |

16. What is your age group?

1. 18-24 years
2. 25-34 years
3. 35-44 years
4. 45-54 years
5. 55-70 years
6. Above 70 years

17. What is your gender?

1. Male
2. Female
3. Non-binary/ third gender
4. Prefer not to answer

18. Are you covered by health insurance (your insurance or someone else’s)?

1. Yes
2. No

19. What is your race?

1. White
2. Black or African American
3. Asian
4. American Indian or Alaska Native
5. Native Hawaiian or Other Pacific Islander
6. Some other race

20. What is your ethnicity?

1. Hispanic or Latino
2. Not Hispanic or Latino

21. About how much do you earn every year?

1. Less than $26,000
2. $26,000 to $51,999
3. $52,000 to $78,000
4. Over $78,000
5. Prefer not to answer

22. What is your marital status?

1. Single (never married)
2. Married, or in a domestic partnership
3. Widowed
4. Divorced
5. Separated
6. Prefer not to answer

23 What is the highest degree or level of school you have completed? (If you’re currently enrolled in school, please indicate the highest degree you have received.)

1. 8th grade or less (no high school)
2. 9th - 11th grade (some high school)
3. High school
4. Some college/Associate’s Degree
5. Bachelor’s Degree (BA, BS, etc.)
6. Graduate or GED Graduate or professional degree

24. What is your current employment status?

1. Employed full time
2. Employed part time
3. Unemployed and currently looking for work
4. Unemployed and not currently looking for work
5. Student
6. Retired
7. Homemaker
8. Self-employed
9. Unable to work

25. Where do you have access to the internet: (check all that apply)

1. Home
2. Work
3. Library
4. School
5. Mobile device/cell phone
6. Other, please specify:

26. How often do you use the Internet?

1. Daily
2. Weekly
3. Monthly
4. Less than monthly

27. When was your last healthcare appointment?

1. Less than 6 months
2. About a year ago
3. Less than 3 years ago
4. More than 3 years
